# Supplementary material for: Effects of jogi, Micropogonias undulatus, addition on the production of volatile compounds in baechu-kimchi
Source: PLoS One. 2024 Nov 18;19(11):e0312441. doi: 10.1371/journal.pone.0312441 (PMC11573143; doi:10.1371/journal.pone.0312441)
Supplement: Attachment — Submitted filename: PONE-D-24-29960_reviewer.pdf [file pone.0312441.s001.pdf]

PLOS ONE

Effects of jogi, Micropogonias undulatus, addition on the production of volatile compounds in baechu-kimchi

--Manuscript Draft--

**Manuscript Number:**	PONE-D-24-29960
**Article Type:**	Research Article
**Full Title:**	Effects of jogi, Micropogonias undulatus, addition on the production of volatile compounds in baechu-kimchi
**Short Title:**	Volatile compounds of jogi-baechu-kimchi
**Corresponding Author:**	Do-Won Jeong<br>Dongduk Women's University<br>Seoul, Seoul KOREA, REPUBLIC OF
**Keywords:**	Baechu-kimchi; jogi; fermentation; volatile compound; sulfur-containing compounds
**Abstract:**	Baechu-kimchi is a traditional vegetable fermented food using kimchi cabbage (Brassica rapa) as the main ingredient. A wide variety of ingredients can be used in kimchi depending on the specific region and even household. Although there have been a lot of studies examining various aspects of kimchi, there has been limited research on kimchi with added fish as a minor ingredient. Therefore, in the present work we aimed to assess changes in the volatile compounds of baechu-kimchi with the addition of seafood used as minor ingredients of kimchi. Baechu-kimchi without jogi was used as the control group. Sulfur-containing compounds were the most commonly detected volatile compounds; 9 categories of volatile components were detected in total. Altogether, 30 sulfur compounds were detected, and among them, five sulfur compounds: (E)-1-(methyltrisulfanyl)prop-1-ene, 1-(methyldisulfanyl)-1-methylsulfanylpropane, (methyltetrasulfanyl)methane, 1-(methyldisulfanyl)-1-[(E)-prop-1-enyl]sulfanylpropane, and 1,1-bis(methyldisulfanyl)propane, were found only in jogi-added kimchi, thus confirming the influence of jogi addition. Principal component analysis revealed clear distinctions in the volatile compounds as a result of jogi addition as fermentation progressed. Moreover, when confirming the correlation with microbial populations, it was evident that the differentiation in volatile compounds was more attributable to jogi addition than microbial impact.
**Order of Authors:**	Gawon Lee
	Sojeong Heo
	Junghyun Park
	Jung-Sug Lee
	Do-Won Jeong
**Additional Information:**	
**Question**	**Response**
**Financial Disclosure**<br><br>Enter a financial disclosure statement that describes the sources of funding for the work included in this submission. Review the submission guidelines for detailed requirements. View published research articles from *PLOS ONE* for specific examples.<br><br>This statement is required for submission	Yes

and **will appear in the published article** if the submission is accepted. Please make sure it is accurate.<br><br>**Funded studies**<br>Enter a statement with the following details:<br>• Initials of the authors who received each award<br>• Grant numbers awarded to each author<br>• The full name of each funder<br>• URL of each funder website<br>• Did the sponsors or funders play any role in the study design, data collection and analysis, decision to publish, or preparation of the manuscript?<br><br>Did you receive funding for this work?	
Please add funding details.<br>as follow-up to "**Financial Disclosure**<br><br>Enter a financial disclosure statement that describes the sources of funding for the work included in this submission. Review the submission guidelines for detailed requirements. View published research articles from *PLOS ONE* for specific examples.<br><br>This statement is required for submission and **will appear in the published article** if the submission is accepted. Please make sure it is accurate.<br><br>**Funded studies**<br>Enter a statement with the following details:<br>• Initials of the authors who received each award<br>• Grant numbers awarded to each author<br>• The full name of each funder<br>• URL of each funder website<br>• Did the sponsors or funders play any role in the study design, data collection and analysis, decision to publish, or preparation of the manuscript?<br><br>Did you receive funding for this work?"	This work was supported by a grant (RS-2022-IP322014) of Korea Institute of Planning and Evaluation for Technology in Food, Agriculture and Forestry (IPET) through the High Value-added Food Technology Development Program funded by the Ministry of Agriculture, Food and Rural Affairs (MAFRA), Republic of Korea.

Please select the country of your main research funder (please select carefully as in some cases this is used in fee calculation).<br>as follow-up to "**Financial Disclosure**<br><br>Enter a financial disclosure statement that describes the sources of funding for the work included in this submission. Review the submission guidelines for detailed requirements. View published research articles from *PLOS ONE* for specific examples.<br><br>This statement is required for submission and **will appear in the published article** if the submission is accepted. Please make sure it is accurate.<br><br>**Funded studies**<br>Enter a statement with the following details:<br>• Initials of the authors who received each award<br>• Grant numbers awarded to each author<br>• The full name of each funder<br>• URL of each funder website<br>• Did the sponsors or funders play any role in the study design, data collection and analysis, decision to publish, or preparation of the manuscript?<br><br>Did you receive funding for this work?"	KOREA, REPUBLIC OF - KR
**Competing Interests**<br><br>Use the instructions below to enter a competing interest statement for this submission. On behalf of all authors, disclose any competing interests that could be perceived to bias this work—acknowledging all financial support and any other relevant financial or non-financial competing interests.<br><br>This statement is required for submission and **will appear in the published article** if the submission is accepted. Please make sure it is accurate and that any funding	The authors have declared that no competing interests exist.

Powered by Editorial Manager® and ProduXion Manager® from Aries Systems Corporation

sources listed in your Funding Information later in the submission form are also declared in your Financial Disclosure statement.<br><br>View published research articles from *PLOS ONE* for specific examples.<br><br>**NO authors have competing interests**<br><br>Enter: *The authors have declared that no competing interests exist.*<br><br>**Authors with competing interests**<br><br>Enter competing interest details beginning with this statement:<br><br>*I have read the journal's policy and the authors of this manuscript have the following competing interests: [insert competing interests here]*<br><br>* typeset	
**Ethics Statement**<br><br>Enter an ethics statement for this submission. This statement is required if the study involved:<br><br>• Human participants<br>• Human specimens or tissue<br>• Vertebrate animals or cephalopods<br>• Vertebrate embryos or tissues<br>• Field research<br><br>Write "N/A" if the submission does not require an ethics statement.<br><br>General guidance is provided below. Consult the submission guidelines for detailed instructions. **Make sure that all information entered here is included in the Methods section of the manuscript.**	N/A

Powered by Editorial Manager® and ProduXion Manager® from Aries Systems Corporation

**Format for specific study types**<br><br>**Human Subject Research (involving human participants and/or tissue)**<br>• Give the name of the institutional review board or ethics committee that approved the study<br>• Include the approval number and/or a statement indicating approval of this research<br>• Indicate the form of consent obtained (written/oral) or the reason that consent was not obtained (e.g. the data were analyzed anonymously)<br><br>**Animal Research (involving vertebrate animals, embryos or tissues)**<br>• Provide the name of the Institutional Animal Care and Use Committee (IACUC) or other relevant ethics board that reviewed the study protocol, and indicate whether they approved this research or granted a formal waiver of ethical approval<br>• Include an approval number if one was obtained<br>• If the study involved *non-human primates*, add *additional details* about animal welfare and steps taken to ameliorate suffering<br>• If anesthesia, euthanasia, or any kind of animal sacrifice is part of the study, include briefly which substances and/or methods were applied<br><br>**Field Research**<br><br>Include the following details if this study involves the collection of plant, animal, or other materials from a natural setting:<br>• Field permit number<br>• Name of the institution or relevant body that granted permission	
**Data Availability**<br><br>Authors are required to make all data underlying the findings described fully available, without restriction, and from the time of publication. PLOS allows rare exceptions to address legal and ethical concerns. See the PLOS Data Policy and FAQ for detailed information.	Yes - all data are fully available without restriction

A Data Availability Statement describing where the data can be found is required at submission. Your answers to this question constitute the Data Availability Statement and **will be published in the article**, if accepted.<br><br>**Important:** Stating 'data available on request from the author' is not sufficient. If your data are only available upon request, select 'No' for the first question and explain your exceptional situation in the text box.<br><br>Do the authors confirm that all data underlying the findings described in their manuscript are fully available without restriction?	
**Describe where the data may be found in full sentences. If you are copying our sample text, replace any instances of XXX with the appropriate details.**<br><br>• If the data are **held or will be held in a public repository**, include URLs, accession numbers or DOIs. If this information will only be available after acceptance, indicate this by ticking the box below. For example: *All XXX files are available from the XXX database (accession number(s) XXX, XXX.).*<br>• If the data are all contained **within the manuscript and/or Supporting Information files**, enter the following: *All relevant data are within the manuscript and its Supporting Information files.*<br>• If neither of these applies but you are able to provide **details of access elsewhere**, with or without limitations, please do so. For example:<br><br>*Data cannot be shared publicly because of [XXX]. Data are available from the XXX Institutional Data Access / Ethics Committee (contact via XXX) for researchers who meet the criteria for access to confidential data.*<br><br>*The data underlying the results presented in the study are available from (include the name of the third party*	All relevant data are within the manuscripts.

and contact information or URL).<br>• This text is appropriate if the data are owned by a third party and authors do not have permission to share the data.<br><br>* typeset	
Additional data availability information:	

Powered by Editorial Manager® and ProduXion Manager® from Aries Systems Corporation

Manuscript

Click here to access/download;Manuscript;240719 manuscript for jogi volatile compound v3.docx

Submitted to the *PLoS ONE*

Effects of *jogi*, *Micropogonias undulatus,* addition on the production of volatile compounds in *baechu-kimchi*

Gawon Lee[1], Sojeong Heo[1], Junghyun Park[1], Jung-Sug Lee[2], Do-Won Jeong[1,*]

[1] *Department of Food and Nutrition, Dongduk Women's University, Seoul 02748, Republic of Korea*

[2] *Department of Food and Nutrition, Kookmin University, Seoul 02707, Republic of Korea*

Running title: Volatile compounds of *jogi-baechu-kimchi*

Corresponding authors

D.-W.J. Phone: +82-2-940-4463; Email: jeongdw@dongduk.ac.kr

Abstract

Baechu-kimchi is a traditional vegetable fermented food using kimchi cabbage (*Brassica rapa*) as the main ingredient. A wide variety of ingredients can be used in kimchi depending on the specific region and even household. Although there have been a lot of studies examining various aspects of kimchi, there has been limited research on kimchi with added fish as a minor ingredient. Therefore, in the present work we aimed to assess changes in the volatile compounds of *baechu-kimchi* with the addition of seafood used as minor ingredients of kimchi. *Baechu-kimchi* without *jogi* was used as the control group. Sulfur-containing compounds were the most commonly detected volatile compounds; 9 categories of volatile components were detected in total. Altogether, 30 sulfur compounds were detected, and among them, five sulfur compounds: (*E*)-1-(methyltrisulfanyl)prop-1-ene, 1-(methyldisulfanyl)-1-methylsulfanylpropane, (methyltetrasulfanyl)methane, 1-(methyldisulfanyl)-1-[(*E*)-prop-1-enyl]sulfanylpropane, and 1,1-bis(methyldisulfanyl)propane, were found only in *jogi*-added kimchi, thus confirming the influence of *jogi* addition. Principal component analysis revealed clear distinctions in the volatile compounds as a result of *jogi* addition as fermentation progressed. Moreover, when confirming the correlation with microbial populations, it was evident that the differentiation in volatile compounds was more attributable to *jogi* addition than microbial impact.

Keywords: *Baechu-kimchi*, *jogi*, fermentation, volatile compound, sulfur-containing compounds

Introduction

Kimchi using *baechu* as a major ingredient is a naturally fermented food for which Codex International Food Standards (CODEX STAN 223-2001) were established in July 2001; *baechu* is kimchi cabbage (CODEX classification No. VB 0467) of the *Brassica rapa* vegetables [1]. *Baechu-kimchi* made with kimchi cabbage varies according to both the minor ingredients used and the region [2, 3]. For example, in coastal areas, seafood is typically added as a minor ingredient when making kimchi [4-7].

Kimchi is a fermented food that features rich tastes and different textures that are imparted through fermentation from raw materials such as *baechu* [8]. During the fermentation process, kimchi forms bacterial communities, which change the ingredients of the raw materials because of the enzymes and metabolites they produce [9-12]. The main bacteria formed in fermented kimchi are lactic acid bacteria, which produce organic acids, amino acids, or volatile compounds during the fermentation process. Lactic acid bacteria also contribute to the sensory properties of fermented *baechu-kimchi* [13, 14]. During the appropriate ripening period when kimchi is properly fermented, it has excellent sensory properties, but after the appropriate ripening period, the sour taste becomes stronger, the tissue becomes softer, and the composition or content of the volatile ingredients changes [8,15,16].

To date, there have been several studies reporting on the volatile compounds of kimchi. These include studies examining changes in the volatile compounds of long-term fermented *baechu-kimchi* [17], changes in the volatile compounds of mustard leaf (*Brassica juncea*) kimchi [18], changes in the volatile compounds of *Angelica keiskei* kimchi [19], changes in the volatile compounds of *Gamdongchotmoo* kimchi [20], changes in the volatile odor components of kimchi as a result of heating [21], and changes in the volatile flavor

compounds in kimchi due to the addition of different ingredients (green onions, garlic, ginger, red pepper powder) [22]. Although there has been a study reporting on the effect that the addition of salted fish has on volatile compounds, there has been no research examining the effect on volatile compounds due to the addition of seafood [23].

In previous studies, we confirmed the effect that the addition of *jogi*—which is typically added into kimchi in Gyeongsangbuk-do—had on the bacterial communities of kimchi [24, 25]. Contrary to our assumptions that the protein content of fish would lead to many protein-using bacteria, there was no significant change in bacterial communities during kimchi fermentation. Other experiments also confirmed that the addition of seafood, *galchi* (*Trichiurus lepturus*), *gul* (*Crassostrea gigas*), *jeonbok* (*Nordotis discus discus*), *hongeu* (*Okamejei kenojei*), and *myeongtae* (*Theragra chalcogramma*), did not affect the microbial communities of kimchi [24, 26]. Another study showed that the addition of *jogi* into kimchi did not have significant effects on organic acid production [27]; however, the amino acid content in the early stages of fermentation was different. It was confirmed that the contents of glutamic acid and aspartic acid, which are savory ingredients, were significantly different immediately after the kimchi was prepared [27]. Altogether, the results confirmed that *jogi* addition did not significantly affect organic acids or bacterial communities, but that it did affect amino acid content. Nevertheless, there has still been insufficient research examining the effect of *jogi* addition on the sensory properties of kimchi. Therefore, this experiment was intended to confirm the effect of *jogi* addition on the volatile compounds that are produced during kimchi fermentation.

Materials and methods

Kimchi samples

The same kimchi that was used in the previous experiment [24,25,27] was used in the current work. Briefly, it is a naturally fermented *baechu-kimchi* (control group) that is manufactured in Sangju, Gyeongsangbuk-do, Republic of Korea; *jogi-baechu-kimchi* was this kimchi with the addition of *jogi* to approximately 5% of the total volume. Kimchi was fermented over 20 days at 10 °C after the initial preparation. Kimchi was sampled every 5 days and stored -80 °C until it was analyzed.

Analysis of volatile compounds of kimchi

One gram of kimchi sample was mixed with 3.25 mL of water and 0.15 g of NaCl in a 15 mL vial with a tan polytetrafluoroethylene/silicone septum (tan PTFE/silicone septum; Supelco, Bellefonte, PA, USA). The sample prepared in this way was then kept at 60 °C for 30 min. The volatile compounds were absorbed onto a 50/30 μm divinylbenzene/carboxen/polydimethylsiloxane fiber (DVB/CAR/PDMS fiber; Supelco) for 15 min, and then eluted at 220 °C. The volatile compounds were transferred through the MS transfer line at 230 °C (ISQ 7000™ Single Quadrupole GC-MS system; Thermo Fisher Scientific, USA). Separation was performed on a TG-WAXMS GC column (30 m length × 0.25 mm i.d. × 0.25 μm film thickness; Thermo Fisher Scientific). The oven program was held at 40 °C for 3 min, raised at 2 °C/min to 150 °C and then held at 150 °C for 10 min, and raised again at 4 °C/min to 200°C and held at 200°C for 10 min; the carrier gas (He) flow rate was 1.0 mL/min, ionization energy was 70 eV, and the mass scan range was 50-550 m/z.

The internal standard was methyl cinnamate (99%) solution at 1.5 μL of 1,000 ppm. The quantitative data were acquired by comparing the peak area (min x counts) of the experimental value obtained by including 1-fold, 2-fold, and 3-fold internal standards excluding kimchi samples, represented by a linear equation. The analysis of volatile

compounds was conducted in triplicate for each sample.

Correlation between microbial community and volatile compounds in kimchi

PAST 4.10 software was used to visualize the correlation between the microbial community and volatile compounds of kimchi. Spearman's rank correlation was used, and values with $p < 0.05$ were considered to be statistically significant.

Statistical analysis

Duncan's multiple range test following a one-way analysis of variance (ANOVA) was used to evaluate significant differences between average values of volatile compounds. Values with $p < 0.05$ were considered to be statistically significant. To visualize differences between volatile compounds of both kimchi samples, principal component analysis (PCA) was performed using SPSS software v.27 (SPSS Inc., Chicago, IL, USA). Additionally, Pearson correlation coefficient (*r*) was also calculated using SPSS software.

Results and discussion

Composition of volatile compounds in kimchi fermentation

To investigate the impact of *jogi* addition on the production of volatile compounds during the fermentation process of kimchi, volatile compounds were analyzed by GC-MS through SPME extraction on different fermentation days (Fig 1). Kimchi without the addition of *jogi*, *baechu-kimchi*, was used as the control group. The evaluations of total acidity, pH, and microbial count, which are indicators of successful fermentation, were established in a

prior experiment [24, 25]. On the first day of fermentation for the control group, seven types of volatile compounds were identified in total, and as fermentation progressed, acid and isoprenoid compounds were newly added (Fig 1A). The kimchi with *jogi* addition, *jogi-baechu-kimchi*, showed a similar trend, but ketone volatile compounds were maintained on the 20th day of fermentation. It was also observed that there were more volatile compounds related to sulfur in the *jogi*-added kimchi.

Fig 1. Number of volatile compounds (A), and amounts of compounds (B) identified in *baechu-kimchi* and *jogi-baechu-kimchi*. The following number indicates the fermentation time (in days).

Although the number of volatile compounds is higher in *jogi-baechu-kimchi*, when they were quantified, the amounts of volatile compounds were higher in *baechu-kimchi* until the 10th day of fermentation (Fig 1B). On the first day of fermentation, alcohols, aldehydes, isothiocyanates, ketones, nitriles, sulfur-compounds, and terpenes were detected, with the amounts of all compounds aside from alcohol being the same or higher in the *baechu-kimchi* than they were in both kimchi types. On the 10th day of fermentation, isoprenoid compounds not detected on the first day were also identified. Comparing the 10th day to the first day, six categories, excluding alcohol and aldehyde, showed similar or higher compound amounts in the *baechu-kimchi*. However, on the 20th day of fermentation, *jogi-baechu-kimchi* displayed higher amounts of volatile compounds, with a particularly high level of sulfur-compounds detected. Sulfur-compounds are known to be major volatile compounds in fish fermentation, and they are classified as strong-smelling substances because of their low odor threshold; moreover, their quantity increases during fish fermentation [28-30]. On the 20th day of fermentation, additional acid compounds were detected compared with the findings on the

10th day, and in the *baechu-kimchi*, ketone compounds disappeared. Furthermore, on the 20th day of fermentation, only S-compounds and terpene compounds were found in higher amounts in *jogi-baechu-kimchi*.

Effect of *jogi* addition on the production of volatile compounds

The volatile compounds detected in *baechu-kimchi* and *jogi-baechu-kimchi* were individually examined (Table 1). To investigate changes that occurred based on the presence or absence of *jogi* addition, the analysis focused on the differences between the two types of kimchi.

Table 1. Effects of *jogi* addition of *baechu-kimchi* on the production of volatile compounds. All samples were repeated in triplicate to confirm results.

Volatile compounds	RT	RI	*Baechu-kimchi*			*Jogi-baechu-kimchi*		
			1 Day	10 Day	20 Day	1 Day	10 Day	20 Day
Acid								
Acetic acid	26.96	1449			$0.935^{c,A}$			$0.773^{b,A}$
Alcohol								
Hexan-1-ol	20.72	1355			$0.606^{c,A}$	$0.537^{b,B}$	$0.522^{b,B}$	$0.596^{c,A}$
6-Methylhept-5-en-2-ol	27.38	1465			$0.569^{c,A}$			$0.552^{b,A}$
(*E*)-Oct-2-en-1-ol	35.89	1614			$0.560^{b,A}$			$0.578^{c,A}$
2-Phenylethanol	51.25	1906			$0.569^{b,A}$			
4-Methyl-3-propan-2-yldec-1-en-4-ol	75.69	1366	$0.534^{ab,A}$	$0.531^{ab,A}$	$0.545^{b,A}$	$0.527^{a,A}$	$0.527^{a,A}$	$0.532^{ab,A}$
Aldehyde								
(*E*)-Hex-2-enal	12.87	1216	$0.913^{c,A}$	$0.848^{c,A}$		$0.777^{bc,A}$	$0.560^{b,A}$	
(*E*)-Hept-2-enal	18.67	1323	$0.536^{b,A}$		$0.618^{d,A}$	$0.529^{b,A}$	$0.523^{b,B}$	$0.567^{c,B}$
Nonanal	22.87	1391	$0.555^{ab,A}$	$0.551^{ab,A}$	$0.584^{ab,A}$	$0.540^{a,A}$	$0.532^{a,A}$	$0.608^{b,A}$
(2*E*,4*E*)-Hepta-2,4-dienal	28.53	1495	$0.586^{c,A}$	$0.589^{c,A}$		$0.557^{bc,A}$	$0.540^{b,A}$	
Isoprenoid								
(*E*)-4-(2,6,6-Trimethylcyclohexen-1-yl)but-3-en-2-one	52.19	1940		$0.612^{b,A}$	$0.695^{c,A}$		$0.584^{b,A}$	$0.635^{b,A}$
Isothiocyanate								
3-Isothiocyanatoprop-1-ene	21.08	1356	$1.902^{c,A}$	$1.920^{c,A}$	$0.909^{ab,A}$	$1.583^{bc,A}$	$0.736^{a,A}$	$0.918^{ab,A}$
4-Isothiocyanatobut-1-ene	26.55	1435	$5.334^{a,A}$	$5.151^{a,A}$	$10.403^{b,A}$	$3.826^{a,A}$	$3.742^{a,A}$	$6.063^{a,A}$
1-Isothiocyanatononane	34.06	1862		$0.572^{b,A}$	$0.618^{c,A}$			
1-Isothiocyanatoheptane	39.97	1666	$0.543^{c,A}$		$0.571^{d,A}$		$0.523^{b,B}$	
1-Isothiocyanato-3-methylsulfanylpropane	54.33	1975	$0.568^{d,A}$	$0.521^{b,A}$		$0.546^{c,A}$		$0.529^{bc,B}$

1-Isothiocyanato-4-methylsulfanylbutane	61.60	2087	1.578^{b,A}	0.536^{a,A}	0.588^{a,A}	0.906^{a,A}	0.566^{a,A}	0.604^{a,A}
(*Z*)-4-Isothiocyanato-1-methylsulfanylbut-1-ene	61.94	1419	0.707^{c,A}			0.576^{b,B}		
2-Isothiocyanatoethylbenzene	67.42	2234	3.464^{b,A}	1.626^{a,A}	3.675^{b,A}	1.524^{a,B}	1.472^{a,A}	2.070^{a,A}
1-Isothiocyanato-5-methylsulfanylpentane	69.96	2242	1.088^{b,A}	0.561^{a,A}	0.657^{a,A}	0.774^{a,A}	0.710^{a,B}	0.668^{a,A}
Ketone								
Undecan-2-one	34.75	1598	0.543^{bc,A}	0.535^{b,A}		0.535^{b,A}	0.527^{b,A}	0.562^{c,B}
Nitrile								
Hex-5-enenitrile	20.03	858	0.661^{a,A}	0.602^{a,A}	0.930^{b,A}	0.672^{a,A}	0.562^{a,A}	0.830^{b,A}
5-Methylsulfanylpentanenitrile	51.87	1851	0.541^{b,A}			0.536^{b,A}		
3-(Thiiran-2-yl)propanenitrile	52.20	1006	0.754^{b,A}			0.720^{b,A}		
3-Phenylpropanenitrile	57.11	2041	1.428^{c,A}	0.714^{a,A}	1.160^{bc,A}	0.976^{ab,A}	0.723^{a,A}	0.983^{ab,A}
6-Methylsulfanylhexanenitrile	58.01	2089	0.614^{c,A}	0.534^{a,A}	0.547^{ab,A}	0.591^{bc,A}	0.556^{ab,A}	0.588^{bc,A}
S compound								
3-Methylsulfanylprop-1-ene	5.21	956	0.595^{bc,A}	0.583^{bc,A}		0.573^{bc,A}	0.549^{b,A}	0.650^{c,B}
(Methyldisulfanyl)methane	6.71	1077	5.390^{c,A}	1.681^{a,A}	0.997^{a,A}	3.747^{b,A}	1.333^{a,A}	1.473^{a,B}
3-Prop-2-enylsulfanylprop-1-ene	10.06	1148	1.095^{ab,A}	1.354^{b,A}	1.908^{c,A}	0.889^{ab,A}	0.790^{a,A}	2.222^{c,A}
1-(Methyldisulfanyl)propane	13.38	1239	0.686^{a,A}	0.786^{ab,A}	0.948^{b,A}	0.664^{a,A}	0.623^{a,A}	1.275^{c,B}
3,4-Dimethylthiophene	14.75	1252		0.562^{bc,A}		0.554^{bc,B}	0.527^{b,B}	0.586^{c,B}
(*E*)-1-(Methyldisulfanyl)prop-1-ene	15.06	1327	0.854^{d,A}	0.660^{abc,A}	0.618^{ab,A}	0.786^{cd,A}	0.574^{a,A}	0.765^{bcd,A}
3-(Methyldisulfanyl)prop-1-ene	16.12	1281	7.940^{c,A}	4.532^{ab,A}	5.623^{bc,A}	5.962^{bc,A}	2.142^{a,A}	7.206^{bc,A}
(Methyltrisulfanyl)methane	21.41	1377	4.849^{b,A}	2.781^{ab,A}	4.339^{b,A}	4.293^{b,A}	2.119^{a,A}	4.697^{b,A}
2-(Prop-2-enyldisulfanyl)propane	24.64	1076	0.816^{a,A}	1.214^{ab,A}	1.700^{b,A}	0.771^{a,A}	0.723^{a,A}	2.364^{c,A}
1-[[(*E*)-Prop-1-enyl]disulfanyl]propane	25.02	1118	0.575^{a,A}	0.570^{a,A}	0.575^{a,A}	0.557^{a,A}	0.522^{a,A}	0.699^{b,B}
3-[(Prop-2-en-1-yl)disulfanyl]prop-1-ene	27.78	1475	9.040^{a,A}	10.723^{ab,A}	17.538^{bc,A}	7.374^{a,A}	5.103^{a,A}	19.582^{c,A}
(*E*)-1-(Prop-2-enyldisulfanyl)prop-1-ene	28.04	1103	4.656^{bc,A}	2.487^{ab,A}	3.381^{abc,A}	2.944^{abc,A}	1.463^{a,A}	5.110^{c,A}
3H-1,2-Dithiole	29.89	1510	0.978^{c,A}	0.756^{a,A}	0.795^{ab,A}	0.779^{ab,A}	0.621^{a,B}	0.952^{bc,A}
1-(Methyltrisulfanyl)propane	30.01	1531			0.875^{c,A}	0.600^{b,B}	0.566^{b,B}	0.989^{c,A}
4-Ethyl-5-methyl-1,3-thiazole	31.15	1412	11.168^{abc,A}	7.757^{ab,A}	17.390^{c,A}	5.278^{a,A}	6.269^{a,A}	13.655^{bc,A}
3-(Methyltrisulfanyl)prop-1-ene	33.66	1593	6.273^{bc,A}	3.699^{ab,A}	4.902^{abc,A}	5.760^{bc,A}	2.395^{a,A}	7.090^{c,A}
(*Z*)-1-(Methyltrisulfanyl)prop-1-ene	34.14	1179	0.617^{abc,A}	0.570^{ab,A}	0.593^{abc,A}	0.652^{bc,A}	0.543^{a,A}	0.680^{c,A}
(*E*)-1-(Methyltrisulfanyl)prop-1-ene	34.33	1586					0.539^{b,B}	0.654^{c,B}
(Methyldisulfanyl)-methylsulfanylmethane	37.53	1650	0.667^{c,A}	0.532^{a,A}	0.538^{a,A}	0.602^{b,A}	0.542^{a,A}	0.545^{a,A}
1,2,5,6,7,8-Hexahydropyrrolizine-3-thione	37.84	1222	0.612^{a,A}	0.575^{a,A}	0.625^{a,A}	0.572^{a,A}	0.573^{a,A}	0.607^{a,A}
1-(Propyltrisulfanyl)propane	37.94	1703			0.560^{b,A}			0.563^{b,A}
1-(Methyldisulfanyl)-1-methylsulfanylpropane	40.84	1207						0.559^{b,B}
3-Ethenyl-3,6-dihydrodithiine	41.34	1750	0.715^{b,A}	0.561^{a,A}	0.608^{ab,A}	0.621^{ab,A}	0.533^{a,A}	0.611^{ab,A}
(Methyltetrasulfanyl)methane	41.63	1746				0.584^{d,B}	0.530^{b,B}	0.550^{c,B}
3-(Prop-2-enyltrisulfanyl)prop-1-ene	44.52	1805	1.674^{bc,A}	1.182^{ab,A}	1.514^{abc,A}	1.412^{abc,A}	0.838^{a,A}	1.959^{c,A}
2-Ethenyl-4H-1,3-dithiine	47.15	1857	0.737^{b,A}	0.659^{ab,A}	0.768^{b,A}	0.646^{ab,A}	0.583^{a,A}	0.747^{b,A}
Methylsulfanyl(methylsulfinyl)methane	48.41	1042	0.566^{d,A}			0.541^{c,A}	0.522^{b,B}	0.535^{bc,B}
1-(Methyldisulfanyl)-1-[(*E*)-prop-1-enyl]sulfanylpropane	48.83	1413					0.523^{b,B}	0.550^{c,B}
1,1-Bis(methyldisulfanyl)propane	56.65	1483.4				0.531^{b,B}		0.536^{b,B}
1,3-Dithiole-2-thione	79.14	1172	0.547^{b,A}	0.526^{a,A}	0.550^{b,A}	0.524^{a,B}	0.521^{a,A}	0.551^{b,A}
Terpene								
3,7-Dimethylocta-1,6-dien-3-ol	32.35	1547	0.568^{a,A}	0.570^{a,A}	0.686^{b,A}	0.551^{a,A}	0.546^{a,A}	0.657^{b,A}
(1,7,7-Trimethyl-2-bicyclo[2.2.1]heptanyl) acetate	33.20	1277	0.538^{b,A}	0.530^{ab,A}	0.535^{ab,A}	0.535^{ab,A}	0.522^{a,A}	0.543^{b,A}
2,6,6-Trimethylcyclohexene-1-carbaldehyde	35.28	1611			0.734^{b,A}			

1-Methyl-4-(6-methylheptan-2-yl)benzene	38.39	1696					0.527^{b,B}	0.555^{c,B}
(2*Z*)-3,7-Dimethylocta-2,6-dienal	39.10	1680	0.606^{c,A}	0.547^{ab,A}	0.567^{ab,A}	0.575^{bc,A}	0.534^{a,A}	0.582^{bc,A}
2-Methyl-5-(6-methylhept-5-en-2-yl)bicyclo[3.1.0]hex-2-ene	41.24	1384	0.908^{ab,A}	1.196^{b,A}	0.821^{ab,A}	0.752^{a,B}	0.929^{ab,A}	1.651^{c,B}
(4S)-1-Methyl-4-(6-methylhepta-1,5-dien-2-yl)cyclohexene	41.54	1727	0.941^{bcd,A}	0.774^{abc,A}	1.064^{d,A}	0.742^{ab,B}	0.654^{a,A}	0.956^{cd,A}
(3*E*,6*E*)-3,7,11-Trimethyldodeca-1,3,6,10-tetraene	43.17	1746	0.644^{ab,A}	0.639^{ab,A}	0.723^{bc,A}	0.595^{a,A}	0.593^{a,A}	0.739^{c,A}
3-(6-Methylhept-5-en-2-yl)-6-methylidenecyclohexene	43.74	1772	0.988^{ab,A}	0.988^{ab,A}	1.233^{bc,A}	0.901^{ab,A}	0.759^{a,A}	1.354^{c,A}
1-Methyl-4-(6-methylhept-5-en-2-yl)benzene	44.08	1777	0.920^{ab,A}	0.943^{ab,A}	1.018^{bc,A}	0.796^{ab,B}	0.740^{a,A}	1.209^{c,A}
(2*E*)-3,7-Dimethylocta-2,6-dien-1-ol	48.52	1847	0.705^{ab,A}	0.618^{a,A}	0.801^{b,A}	0.647^{a,A}	0.585^{a,A}	0.788^{b,A}

1) a, b, c, d Different superscripts in a column indicate significant differences at $p<0.05$ according to independent *t*-test.
2) A, B Different superscripts in a row indicate significant differences at $p<0.05$ according to Duncan's multiple range test.

Acetic acid was detected in the acid category, and this was observed in both types of kimchi on the 20th day of fermentation. Acetic acid, which is an organic acid that is known to impart a sour taste to kimchi, has also been observed to increase in quantity as fermentation progressed in previous experiments [31]. Therefore, it can be inferred that this volatile compound is produced during the fermentation process of kimchi and is not influenced by *jogi* addition.

In the alcohol category, five volatile compounds were detected: hexan-1-ol, 6-methylhept-5-en-2-ol, (*E*)-oct-2-en-1-ol, 2-phenylethanol, and 4-methyl-3-propan-2-yldec-1-en-4-ol. Aside from 2-phenylethanol, there were no significant differences in the detected amounts of the compounds, indicating that they are commonly produced during kimchi fermentation. Interestingly, 2-phenylethanol was detected in the control group but not in the *jogi-baechu-kimchi*. Previous studies have shown findings of 2-phenylethanol detection from day 0 to day 30 of fermentation regardless of the addition of fish sauce [32]. However, recent analyses of kimchi using the same SPME fiber (DVB/CAR/PDMS, 50/30 μm, Supelco) did not confirm the presence of 2-phenylethanol [33]. It is therefore speculated that the absence of this volatile compound in *jogi-baechu-kimchi* may be due to sample variations rather than a reduction caused by *jogi* addition, or that it may be a component that is detected later in the

fermentation process.

Four volatile compounds were detected in the aldehyde category, (*E*)-hex-2-enal, (*E*)-hept-2-enal, nonanal, and (2*E*,4*E*)-hepta-2,4-dienal, while in the isoprenoid category, one volatile compound, (*E*)-4-(2,6,6-trimethylcyclohexen-1-yl)but-3-en-2-one, was identified. However, the only statistically significant difference was observed in (*E*)-hept-2-enal, which was found to be higher in *baechu-kimchi*. (*E*)-Hex-2-enal is frequently detected in kimchi fermentation [32], and the decrease in the quantity of (*E*)-hex-2-enal is presumed to be due to the addition of *jogi* affecting this volatile compound.

In the isothiocyanate category, 1-isothiocyanatononane was detected from the mid-fermentation stage in the control group, while (*Z*)-4-isothiocyanato-1-methylsulfanylbut-1-ene was observed only on the first day of fermentation and then disappeared, irrespective of the addition of *jogi*. Isothiocyanates are pungent-odor volatile compounds that are typically detected in the early stages of fermentation, but which tend to decrease as fermentation progresses [34]. A similar trend was observed in this experiment, with the number of isothiocyanates in both *baechu-kimchi* and *jogi-baechu-kimchi* decreasing as fermentation progressed compared with that in the early fermentation stages.

In the ketone category, undecan-2-one was detected. It was present in *baechu-kimchi* until the 10th day, while in *jogi-baechu-kimchi*, the quantity remained similar even on the 20th day. A more thorough investigation as to whether the addition of *jogi* prolongs the presence of the undecan-2-one is needed.

The sulfur-containing compounds (S compounds) exhibited the highest number of volatile compounds. These compounds are known to be generated from the main and minor ingredients used when making kimchi, such as cabbage, radish, red pepper, garlic, green onion, and ginger [35]. In total, 20 S compounds were consistently detected in all samples regardless of the presence of *jogi* addition. However, five S compounds, namely (*E*)-1-

(methyltrisulfanyl)prop-1-ene, 1-(methyldisulfanyl)-1-methylsulfanylpropane, (methyltetrasulfanyl)methane, 1-(methyldisulfanyl)-1-[(*E*)-prop-1-enyl]sulfanylpropane, and 1,1-bis(methyldisulfanyl)propane, were exclusively found in *jogi-baechu-kimchi*. Among these, the four volatile compounds that remain after excluding (methyltetrasulfanyl)methane are also detected in garlic and onion [36-39]. Regarding (methyltetrasulfanyl)methane, it has been identified as a representative volatile compound in shrimp paste and fish [40, 41], suggesting that it might be derived from seafood.

In the terpene category, nine volatile compounds were detected in all samples, regardless of the fermentation period or *jogi* addition. 2,6,6-Trimethylcyclohexene-1-carbaldehyde was found only on the 20th-day for *baechu-kimchi*, while 1-methyl-4-(6-methylheptan-2-yl)benzene was detected only in the late-stages of fermentation of of *jogi-baechu-kimchi*. Terpenes have previously been detected in large quantities in the early stages of kimchi fermentation, where they have also been shown to decrease after 30 days of fermentation [32]. However, in this experiment, kimchi fermentation was only conducted for 20 days, and there was no significant difference in terpene content. Moreover, sesquiterpenes are relatively less influential in the flavor of kimchi compared with sulfur compounds because of their higher odor threshold levels [32].

The above results confirm that *jogi*-addition affects the production of sulfur compounds among volatile compounds. Specifically, five volatile compounds, (*E*)-1-(methyltrisulfanyl)prop-1-ene, 1-(methyldisulfanyl)-1-methylsulfanylpropane, (methyltetrasulfanyl)methane, 1-(methyldisulfanyl)-1-[(*E*)-prop-1-enyl]sulfanylpropane, and 1,1-bis(methyldisulfanyl)propane, are observed exclusively in *jogi-baechu-kimchi*, indicating the influence of *jogi*.

PCA and screening of volatile profiles of kimchi fermentation

To visually assess the types and quantities of volatile compounds produced in *jogi-baechu-kimchi* and *baechu-kimchi*, principal component analysis (PCA) was conducted using SPSS (Fig 2). Among the nine volatile compounds categories, the most detected S compounds are generally distributed, primarily in the positive position based on PC1 (Fig 2A). The volatile compounds produced in both types of kimchi were found to be positioned in the first or second quadrant on the first day and then shifted to the second or third quadrant on the 10th day, thus indicating a negative correlation with PC1 (Fig 2B). However, *jogi-baechu-kimchi* can be observed to be positioned in the region where sulfur compounds are more prevalent. Consequently, there is a slight difference in the amount or type of volatile compounds present at the early fermentation stages, showing a clear distinction on the 20th day of fermentation.

Fig 2. Principal component analysis loading (A) and factor scores (B), and Pearson correlation coefficient (C) of 67 volatile compounds identified from two types of *baechu-kimchi*. In (A), volatile compounds are displayed in different colors for each category. In (B), the number means the number of days of fermentation. In (C), * and ** represent statistical significance at the 0.05 and 0.01 levels, respectively. Abbreviations: AC, acid; ALC, alcohol; ALD, aldehyde; I, isoprenoid; IS, isothiocyanate; K, ketone; N, nitrile; S, S compound; T, terpene. JBK, *jogi-baechu-kimchi*; BK, *baechu-kimchi*; the following number indicates the fermentation day. AC1, acetic acid; ALC1, hexan-1-ol; ALC2, 6-methylhept-5-en-2-ol; ALC3, (*E*)-oct-2-en-1-ol; ALC4, 2-phenylethanol; ALC5, 4-methyl-3-propan-2-yldec-1-en-4-ol; ALD1, (*E*)-hex-2-enal; ALD2, (*E*)-hept-2-enal; ALD3, nonanal; ALD4, (2*E*,4*E*)-hepta-2,4-dienal; I1, (*E*)-4-(2,6,6-trimethylcyclohexen-1-yl)but-3-en-2-one; IS1, 3-isothiocyanatoprop-

1-ene; IS2, 4-isothiocyanatobut-1-ene; IS3, 1-isothiocyanatononane; IS4, 1-isothiocyanatoheptane; IS5, 1-isothiocyanato-3-methylsulfanylpropane; IS6, 1-isothiocyanato-4-methylsulfanylbutane; IS7, (*Z*)-4-isothiocyanato-1-methylsulfanylbut-1-ene; IS8, 2-isothiocyanatoethylbenzene; IS9, 1-isothiocyanato-5-methylsulfanylpentane; K1, undecan-2-one; N1, hex-5-enenitrile; N2, 5-methylsulfanylpentanenitrile; N3, 3-(thiiran-2-yl)propanenitrile; N4, 3-phenylpropanenitrile; N5, 6-methylsulfanylhexanenitrile; S1, 3-methylsulfanylprop-1-ene; S2, (methyldisulfanyl)methane; S3, 3-prop-2-enylsulfanylprop-1-ene; S4, 1-(methyldisulfanyl)propane; S5, 3,4-dimethylthiophene; S6, (*E*)-1-(methyldisulfanyl)prop-1-ene; S7, 3-(methyldisulfanyl)prop-1-ene; S8, (methyltrisulfanyl)methane; S9, 2-(prop-2-enyldisulfanyl)propane; S10, 1-[[(*E*)-prop-1-enyl]disulfanyl]propane; S11, 3-[(prop-2-en-1-yl)disulfanyl]prop-1-ene; S12, (*E*)-1-(prop-2-enyldisulfanyl)prop-1-ene; S13, 3H-1,2-dithiole; S14, 1-(methyltrisulfanyl)propane; S15, 4-ethyl-5-methyl-1,3-thiazole; S16, 3-(methyltrisulfanyl)prop-1-ene; S17, (*Z*)-1-(methyltrisulfanyl)prop-1-ene; S18, (*E*)-1-(methyltrisulfanyl)prop-1-ene; S19, (methyldisulfanyl)-methylsulfanylmethane; S20, 1,2,5,6,7,8-hexahydropyrrolizine-3-thione; S21, 1-(propyltrisulfanyl)propane; S22, 1-(methyldisulfanyl)-1-methylsulfanylpropane; S23, 3-ethenyl-3,6-dihydrodithiine; S24, (methyltetrasulfanyl)methane; S25, 3-(prop-2-enyltrisulfanyl)prop-1-ene; S26, 2-ethenyl-4H-1,3-dithiine; S27, methylsulfanyl(methylsulfinyl)methane; S28, 1-(methyldisulfanyl)-1-[(*E*)-prop-1-enyl]sulfanylpropane; S29, 1,1-bis(methyldisulfanyl)propane; S30, 1,3-dithiole-2-thione; T1, 3,7-dimethylocta-1,6-dien-3-ol; T2, (1,7,7-trimethyl-2-bicyclo[2.2.1]heptanyl) acetate; T3, 2,6,6-trimethylcyclohexene-1-carbaldehyde; T4, 1-methyl-4-(6-methylheptan-2-yl)benzene; T5, (2*Z*)-3,7-dimethylocta-2,6-dienal; T6, 2-methyl-5-(6-methylhept-5-en-2-yl)bicyclo[3.1.0]hex-2-ene; T7, (4S)-1-methyl-4-(6-methylhepta-1,5-dien-2-yl)cyclohexene; T8, (3*E*,6*E*)-3,7,11-trimethyldodeca-1,3,6,10-tetraene; T9, 3-(6-methylhept-5-en-2-yl)-6-

methylidenecyclohexene; T10, 1-methyl-4-(6-methylhept-5-en-2-yl)benzene; T11, (2*E*)-3,7-dimethylocta-2,6-dien-1-ol.

Correlation of volatile compounds and fermentation periods

The correlation between the volatile compounds identified in *jogi-baechu-kimchi* and *baechu-kimchi* and the fermentation dates was analyzed using a heatmap (Fig 3). Volatile compounds such as aldehydes, isothiocyanates, ketones, and nitriles tend to decrease or become undetectable as fermentation progresses. Various prior studies have demonstrated the reduction or absence of these components during fermentation [32, 43-45]. By contrast, acids, alcohols, isoprenoids, sulfur compounds, and terpenes are volatile compounds that are detected as fermentation progresses. Among these, the changes in volatile compounds due to *jogi* addition show significant differences, particularly in sulfur compounds. 3-Prop-2-enylsulfanylprop-1-ene, 1-(methyldisulfanyl)propane, 2-(prop-2-enyldisulfanyl)propane, 1,2,5,6,7,8-hexahydropyrrolizine-3-thione, and 1-(methyldisulfanyl)-1-methylsulfanylpropane are components that showed statistically significant results only in *jogi-baechu-kimchi*. Hence, the changes in volatile compounds due to *jogi* addition and fermentation were confirmed.

Fig 3. Correlation between volatile compounds and fermentation periods.

The components that consistently showed positive results in volatile compounds are acetic acid, 6-methylhept-5-en-2-ol, (*E*)-oct-2-en-1-ol, 4-isothiocyanatobut-1-ene, 1-(methyltrisulfanyl)propane, 1-(propyltrisulfanyl)propane, 3,7-dimethylocta-1,6-dien-3-ol, and (3*E*,6*E*)-3,7,11-trimethyldodeca-1,3,6,10-tetraene. These eight volatile compounds were

commonly detected on the 20th day of fermentation. Among them, acetic acid, (*E*)-oct-2-en-1-ol, 1-(methyltrisulfanyl)propane, 1-(propyltrisulfanyl)propane, 3,7-dimethylocta-1,6-dien-3-ol, and (3*E*,6*E*)-3,7,11-trimethyldodeca-1,3,6,10-tetraene were also identified in previous studies examining kimchi volatile compounds [32, 35, 43, 46]. Additionally, these five volatile compounds showed a positive correlation with each other (Fig 2C). Therefore, these components could be considered to be potential biomarkers for confirming the progress of the fermentation.

To confirm the correlation among volatile compounds, Pearson correlation was analyzed as a heatmap (Fig 2C). While the results in Fig 3 were to confirm the correlation between fermentation period and volatile compounds, the results in Fig 2C were to confirm the correlation between volatile compounds. To confirm and verify the results in Fig 3, the correlation between volatile compounds was verified using PAST 4 and SPSS software (Fig 2C). 67 volatile compounds could be divided into two categories, 16 of which were negative for the rest and showed positive correlation among themselves. The volatile compounds showing negative correlation are those that are not detected in the later stages of fermentation, unlike those that are generated as fermentation progresses. Additionally, compounds with negative correlations in Fig 3 also exhibit negative correlations in Fig 2C. For example, the volatile compounds that significantly decreased in the later stages of fermentation, such as (*E*)-hex-2-enal, (2*E*,4*E*)-hepta-2,4-dienal, 3-isothiocyanatoprop-1-ene, 1-isothiocyanato-3-methylsulfanylpropane, undecan-2-one, and 3-methylsulfanylprop-1-ene, belong to the group showing negative values in Fig 2C. Therefore, the correlation between increasing and decreasing volatile compounds as fermentation progresses can be identified.

Correlation of volatile compounds and microbial community in

kimchi

In the kimchi used in this experiment, as analyzed in a previous study investigating microbial communities, *jogi* has been found to initially have a minor impact on the microbial community of cabbage kimchi but almost no influence after 10 days of fermentation [24, 25]. Nevertheless, we aimed to analyze the correlation between the microbial community and volatile compounds of kimchi (Fig 4). Because *jogi* addition had a minimal impact on the microbiota, we analyzed the correlation between the volatile compounds detected in all samples, regardless of the addition of seafood, and the microbial communities separated through a cultivation-dependent method, with the results yielding $p < 0.05$ values. Among the nine categories of volatile compounds, only five categories showed a correlation with the microbial community. *Bacillus* species exhibited a positive correlation with isothiocyanates, nitriles, and S compounds, *Lactilactobacillus sakei* showed a positive correlation with isoprenoids and isothiocyanates, and *Mammaliicoccus sciuri* had a positive correlation with isothiocyanates and S compounds. In contrast, *Weissella cibaria* and *Weissella koreensis*, both of which belong to the *Weissella* genus, exhibited a negative correlation with S compounds and terpenes. Meanwhile, the *Leuconostoc* genus, which was represented by three different species, showed distinct correlations, thus suggesting the existence of inter-species differences.

Fig 4. Correlation between volatile compounds and microbial community in *baechu-kimchi* and *jogi-baechu-kimchi*.

Interestingly, among the volatile compounds that are thought to be related to microorganisms, excluding (*E*)-4-(2,6,6-trimethylcyclohexen-1-yl)but-3-en-2-one and 4-

isothiocyanatobut-1-ene, most of the volatile compounds were primarily detected in the early stages of fermentation. Moreover, the microorganisms that were positively correlated with these volatile compounds were found to proportionately decrease as fermentation progressed. These results suggest that the rich volatile compounds derived from various raw materials in the early stages of fermentation stabilize the microbial community, thus allowing the volatile compounds to harmonize as fermentation progresses. The results also indicate that the impact of lactic acid bacteria on volatile compounds is lower than that of the *Bacillus* genus. However, the (*E*)-4-(2,6,6-trimethylcyclohexen-1-yl)but-3-en-2-one and 4-isothiocyanatobut-1-ene detected in the late stages of fermentation show a positive correlation with *Lb. sakei*, indicating its contribution to the volatile compounds in the final stages of fermentation.

In conclusion, previous research has confirmed that the addition of seafood influenced the amino acid content more than microbial communities, thereby resulting in regional diversity. In this study, we further established that *jogi* addition affects the production of sulfur compounds in cabbage kimchi. The specific mechanisms through which sulfur compounds are generated should be further investigated in future research.

Author Contributions

Conceptualization: Gawon Lee, Sojeong Heo, Do-Won Jeong

Data curation: Gawon Lee, Sojeong Heo

Formal analysis: Gawon Lee, Sojeong Heo, Junghyun Park

Funding acquisition: Do-Won Jeong

Investigation: Gawon Lee, Junghyun Park, Jung-Sug Lee, Do-Won Jeong

Methodology: Gawon Lee, Sojeong Heo, Junghyun Park, Jung-Sug Lee

Project administration: Do-Won Jeong

Supervision: Do-Won Jeong

Writing – original draft: Gawon Lee, Sojeong Heo, Do-Won Jeong

Writing – review & editing: Gawon Lee, Park, Jung-Sug Lee, Do-Won Jeong

References

1. Im MH. Review of Codex Alimentarius and comparison between the US and Korean food classifications for pesticide residues of the US and Korea. J. Korean Soc. Appl. Biol. Chem. 2013; 56: 483-95. https://doi.org/10.1007/s13765-013-3182-x
2. Korea Agro-Fisheries & Food Trade Corporation. Kimchi industry survey analysis report (in Korean). Korea Rural Economic Institute, 2022;10. https://www.atfis.or.kr/home/pdf/view.do?path=/board/202204/6434aee0-8b54-401d-b943-1f0417d1ccf5.pdf (accessed October 2023).
3. Moon GS, Song YS, Jeon YS. A study of famous traditional kimchi in Pusan and near Pusan area. Korean J. Food Cook. Sci. 1996; 12(1): 74-81.
4. Jung YK, Oh SH, Kim SD. Fermentation and quality characteristics of *Kwamaegi* added kimchi. Korean J. Food Preserv. 2007; 14(5): 526-30.
5. Jang MS, Jung KE, Yun JU, Nam KH. Production and fermentation characteristics of seafood kimchi started with *Leuconostoc mesenteriodes* SK-1 isolated from octopus baechu kimchi. Korean J. Food Preserv. 2016; 23(7): 1050-7. https://doi.org/10.11002/kjfp.2016.23.7.1050
6. Lee MA, Seo HY, Yang JH, Jang MS. Effect of squid and octopus on the quality characteristics of kimchi during fermentation. J. Korean Soc. Food Sci. Nutr. 2013; 42(12): 2004-11. https://doi.org/10.3746/jkfn.2013.42.12.2004
7. Woo M, Choi JR, Kim M, Jang MS, Cho EJ, Song YO. Physicochemical characteristics of seafood-added kimchi during fermentation and its sensory properties. J. Korean Soc. Food Sci. Nutr. 2012; 41(12); 1771-7. https://doi.org/10.3746/jkfn.2012.41.12.1771
8. Surya R, Lee AGY. Exploring the philosophical values of kimchi and kimjang culture. J. Ethn. Foods. 2022; 9: 20. https://doi.org/10.1186/s42779-022-00136-5

9. Hong SW, Choi YJ, Lee HW, Yang JH, Lee MA. Microbial community structure of Korean cabbage kimchi and ingredients with denaturing gradient gel electrophoresis. J. Microbiol. Biotechnol. 2016; 26(6): 1057-62. https://doi.org/10.4014/jmb.1512.12035 PMID: 26907755
10. Jung JY, Lee SH, Jeon CO. Microbial community dynamics during fermentation of doenjang-meju, traditional Korean fermented soybean. Int. J. Food Microbiol. 2014; 185: 112-20. https://doi.org/10.1016/j.ijfoodmicro.2014.06.003 PMID: 24960292
11. Lee KW, Shim JM, Kim DW, Yao Z, Kim JA, Kim HJ, et al. Effects of different types of salts on the growth of lactic acid bacteria and yeasts during kimchi fermentation. Food Sci. Biotechnol. 2018; 27: 489-98. https://doi.org/10.1007/s10068-017-0251-7 PMID: 30263773
12. Park EJ, Chun J, Cha CJ, Park WS, Jeon CO, Bae JW. Bacterial community analysis during fermentation of ten representative kinds of kimchi with barcoded pyrosequencing. Food Microbiol. 2012; 30(1): 197-204. https://doi.org/10.1016/j.fm.2011.10.011 PMID: 22265301
13. Hwang H, Lee JH. Characterization of arginine catabolism by lactic acid bacteria isolated from kimchi. Molecules, 2018; 23(11): 3049. https://doi.org/10.3390/molecules23113049 PMID: 30469432
14. Jung MJ, Kim J, Lee SH, Whon TW, Sung H, Bae JW, et al. Role of combinated lactic acid bacteria in bacterial, viral, and metabolite dynamics during fermentation of vegetable food, kimchi. Food Res. Int. 2022; 157: 111261. https://doi.org/10.1016/j.foodres.2022.111261 PMID: 35761573
15. Choi YJ, Lee HW, Yang JH, Park SH, Lee MA. Effects of sweetener on the quality characteristics of radish kimchi during fermentation. Cogent Food Agric. 2023; 9(2): 2286045. https://doi.org/10.1080/23311932.2023.2286045

16. Jang SH, Kim MJ, Lim J, Hong JH. Cross-cultural comparison of consumer acceptability of kimchi with different degree of fermentation. J. Sens. Stud. 2016; 31(2), 124-34. https://doi.org/10.1111/joss.12198
17. Kim JY, Park EY, Kim YS. Characterization of volatile compounds in low-temperature and long-term fermented baechu kimchi. J. Korean Soc. Food Cult. 2006; 21(3): 319-24. https://doi.org/10.7318/KJFC.2006.21.3.319
18. Pyo YH, Kim JS, Hahn YS. Volatile compounds of mustard leaf (*Brassica juncea*) kimchi and their changes during fermentation. Korean J. Food Sci. Technol. 2000; 32(1): 56-61.
19. Chun SS, Cho YS, Shim SY, Shon MY, Choi SH, Lee SR. Changes in chlorphyll contents and volatile compounds of *Angelica keiskei* kimchi during fermentation. Korean J. Food Nutr. 2000; 13(1): 59-65.
20. Yoon MK, Kwon MJ, Lee SM, Kim JW, Cho MS, Lee JM, et al. Characterization of volatile components according to fermentation periods in Gamdongchotmoo kimchi. Korean J. Food Sci. Technol. 2008; 40(5): 497-502.
21. Ko YT, Baik IH. Changes in pH, sensory properties and volatile odor components of kimchi by heating. Korean J. Food Sci. Technol. 2002; 34(6): 1123-6.
22. Ryu JY, Lee HS, Rhee HS. Changes of organic acids and volatile flavor compounds in kimchis fermented with different ingredients. Korean J. Food Sci. Technol. 1984; 16(2): 169-74.
23. Lee HJ, Lee MJ, Choi YJ, Park SJ, Lee MA, Min SG, et al. Free amino acid and volatile compound profiles of jeotgal alternatives and its application to kimchi. Foods, 2021; 10(2): 423. https://doi.org/10.3390/foods10020423 PMID: 33671949
24. Park J, Heo S, Lee G, Hong SW, Jeong DW. Bacterial diversity of baechu-kimchi with seafood based on culture-independent investigations. Food Sci. Biotechnol. 2024; 33: 1661-70. https://doi.org/10.1007/s10068-023-01471-2 PMID: 38623433

25. Park J, Heo S, Na HE, Lee G, Kim T, Sung MH, et al. Culture-dependent and -independent investigations of the effect of addition of *jogi* on the bacterial community of kimchi. Food Biosci. 2023; 54: 102832. https://doi.org/10.1016/j.fbio.2023.102832
26. Kim T, Heo S, Na HE, Lee G, Kim JH, Kwak MS, et al. Bacterial community of galchi-baechu kimchi based on culture-dependent and - independent investigation and selection of starter candidates. J. Microbiol. Biotechnol. 2022; 32(3): 341-7. https://doi.org/10.4014/jmb.2112.12003 PMID: 35001009
27. Park J, Heo S, Lee G, Kim T, Oh SE, Kwak MS, et al. The addition of *jogi*, *Micropogonias undulates*, affects amino acid content in kimchi fermentation. Plos One, 2024; 19(4): e0300249. https://doi.org/10.1371/journal.pone.0300249 PMID: 38573994
28. Ding A, Zhu M, Qian X, Shi L, Huang H, Xiong G, et al. Effect of fatty acids on the flavor formation of fish sauce. LWT - Food Sci. Technol. 2020; 134: 110259. https://doi.org/10.1016/j.lwt.2020.110259
29. Sakpetch P, Benchama O, Masniyom P, Salaipeth L, Kanjan P. Physicochemical characteristics and flavor profiles of fermented fish sauce (budu) during fermentation in commercial manufacturing plant. J. Food Sci. Technol. 2022; 59: 693-702. https://doi.org/10.1007/s13197-021-05064-x PMID: 35153312
30. Udomsil N, Rodtong S, Choi YJ, Hua Y, Yongsawatdigul J. Use of *Tetragenococcus halophilus* as a starter culture for flavor improvement in fish sauce fermentation. J. Agric. Food Chem. 2011; 59(15): 8401-8. https://doi.org/10.1021/jf201953v PMID: 21710980
31. Shim SM, Kim JY, Lee SM, Park JB, Oh SK, Kim YS. Profiling of fermentative metabolites in kimchi: Volatile and non-volatile organic acids. J. Korean Soc. Appl. Biol. Chem. 2012; 55: 463-9. https://doi.org/10.1007/s13765-012-2014-8
32. Cha YJ, Kim H, Cadwallader KR. Aroma-active compounds in kimchi during fermentation. J. Agric. Food Chem. 1998; 46(5): 1944-53.

https://doi.org/10.1021/jf9706991

33. Choi YJ, Yong S, Lee MJ, Park SJ, Yun YR, Park SH, et al. Changes in volatile and non-volatile compounds of model kimchi through fermentation by lactic acid bacteria. LWT - Food Sci. Technol. 2019; 105: 118-26. https://doi.org/10.1016/j.lwt.2019.02.001
34. Lee JH, Lee KT, Kim MR. Effect of gamma-irradiated red pepper powder on the chemical and volatile characteristics of kakdugi, a Korean traditional fermented radish kimchi. J. Food Sci. 2005; 70(7): c441-7. https://doi.org/10.1111/j.1365-2621.2005.tb11466.x
35. Hong SP, Lee EJ, Kim YH, Ahn DU. Effect of fermentation temperature on the volatile composition of kimchi. J. Food Sci. 2016; 81(11): C2623-9. https://doi.org/10.1111/1750-3841.13517 PMID: 27750382
36. Farkas P, Hradský P, Kovác M. Novel flavour components identified in the steam distillate of onion (*Allium cepa* L.). Z. Lebensm. Unters. Forsch. 1992; 195: 459–62. https://doi.org/10.1007/BF01191718
37. Kuo MC, Ho CT. Volatile constituents of the distilled oils of Welsh onions (*Allium fistulosum* L. variety maichuon) and scallions (*Allium fistulosum* L. variety caespitosum). J. Agri. Food Chem. 1992; 40(1): 111-7. https://doi.org/10.1021/jf00013a021
38. Radulović NS, Miltojević AB, Stojković MB, Blagojević PD. New volatile sulfur-containing compounds from wild garlic (*Allium ursinum* L., Liliaceae). Food Res. Int. 2015; 78: 1-10. https://doi.org/10.1016/j.foodres.2015.11.019 PMID: 28433269
39. Wang R, Qiao L, Wang J, Wang J, Zhang N, Chen H, et al. Effect of different vegetable oils on the flavor of fried green onion (*Allium fistulosum* L.) oil. Foods, 2023; 12(7): 1442. https://doi.org/10.3390/foods12071442 PMID: 37048263
40. Fan Y, Yin L, Xue Y, Li Z, Hou H, Xue C. Analyzing the flavor compounds in Chinese traditional fermented shrimp pastes by HS-SPME-GC/MS and electronic nose. J. Ocean Univ. China, 2017; 16: 311-8. https://doi.org/10.1007/s11802-017-3194-y

41. Moreira N, Valente LMP, Castro-Cunha M, Cunha LM, Guedes de Pinho P. Effect of storage time and heat processing on the volatile profile of Senegalese sole (*Solea senegalensis* Kaup, 1858) muscle. Food Chem. 2013; 138(4): 2365-73. https://doi.org/10.1016/j.foodchem.2012.11.135 PMID: 23497897

42. Pereira LSA, Lisboa FLC, Neto JC, Valladão FN, Sena MM. Screening method for rapid classification of psychoactive substances in illicit tablets using mid infrared spectroscopy and PLS-DA. Forensic Sci. Int. 2018; 288: 227-35. https://doi.org/10.1016/j.forsciint.2018.05.001 PMID: 29777946

43. Lee H, Kizito SA, Weese SJ, Craig-Schmidt MC, Lee Y, Wei CI, et al. Analysis of headspace volatile and oxidized volatile compounds in DHA-enriched fish oil on accelerated oxidative storage. J. Food Sci. 2003; 68(7): 2169-77. https://doi.org/10.1111/j.1365-2621.2003.tb05742.x

44. Lee JJ, Choi YJ, Lee MJ, Park SJ, Oh SJ, Yun YR, et al. Effects of combining two lactic acid bacteria as a starter culture on model kimchi fermentation. Food Res. Int. 2020; 136: 109591. https://doi.org/10.1016/j.foodres.2020.109591 PMID: 32846617

45. Taveira M, Fernandes F, Guedes de Pinho P, Andrade PB, Pereira JA, Valentão P. Evolution of *Brassica rapa* var. *rapa* L. volatile composition by HS-SPME and GC/IT-MS. Microchem. J. 2009; 93(2): 140-6. https://doi.org/10.1016/j.microc.2009.05.011

46. Kang JH, Lee JH, Min S, Min DB. Changes of volatile compounds, lactic acid bacteria, pH, and headspace gases in kimchi, a traditional Korean fermented vegetable product. J. Food Sci. 2003; 68(3): 849-54. https://doi.org/10.1111/j.1365-2621.2003.tb08254.x

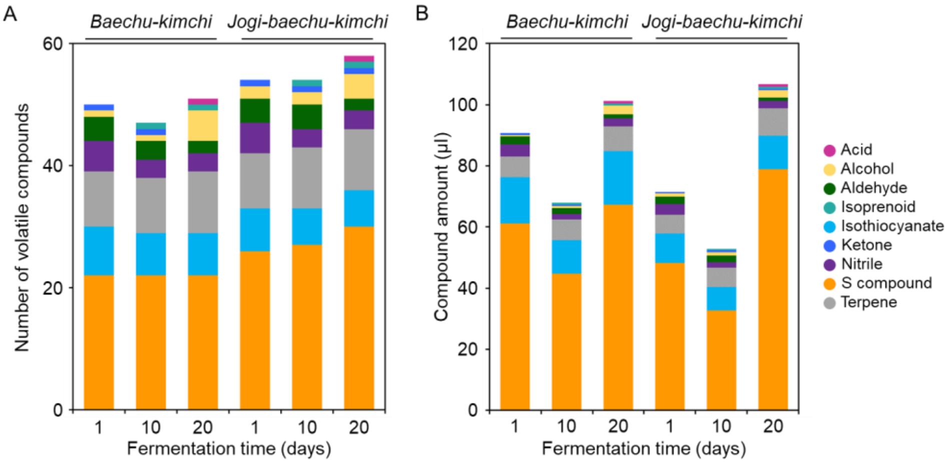

A
Baechu-kimchi
Jogi-baechu-kimchi
Number of volatile compounds
0
20
40
60
1
10
20
1
10
20
Fermentation time (days)
B
Baechu-kimchi
Jogi-baechu-kimchi
Compound amount (μl)
0
20
40
60
80
100
120
1
10
20
1
10
20
Fermentation time (days)
Acid
Alcohol
Aldehyde
Isoprenoid
Isothiocyanate
Ketone
Nitrile
S compound
Terpene

Fig 1.

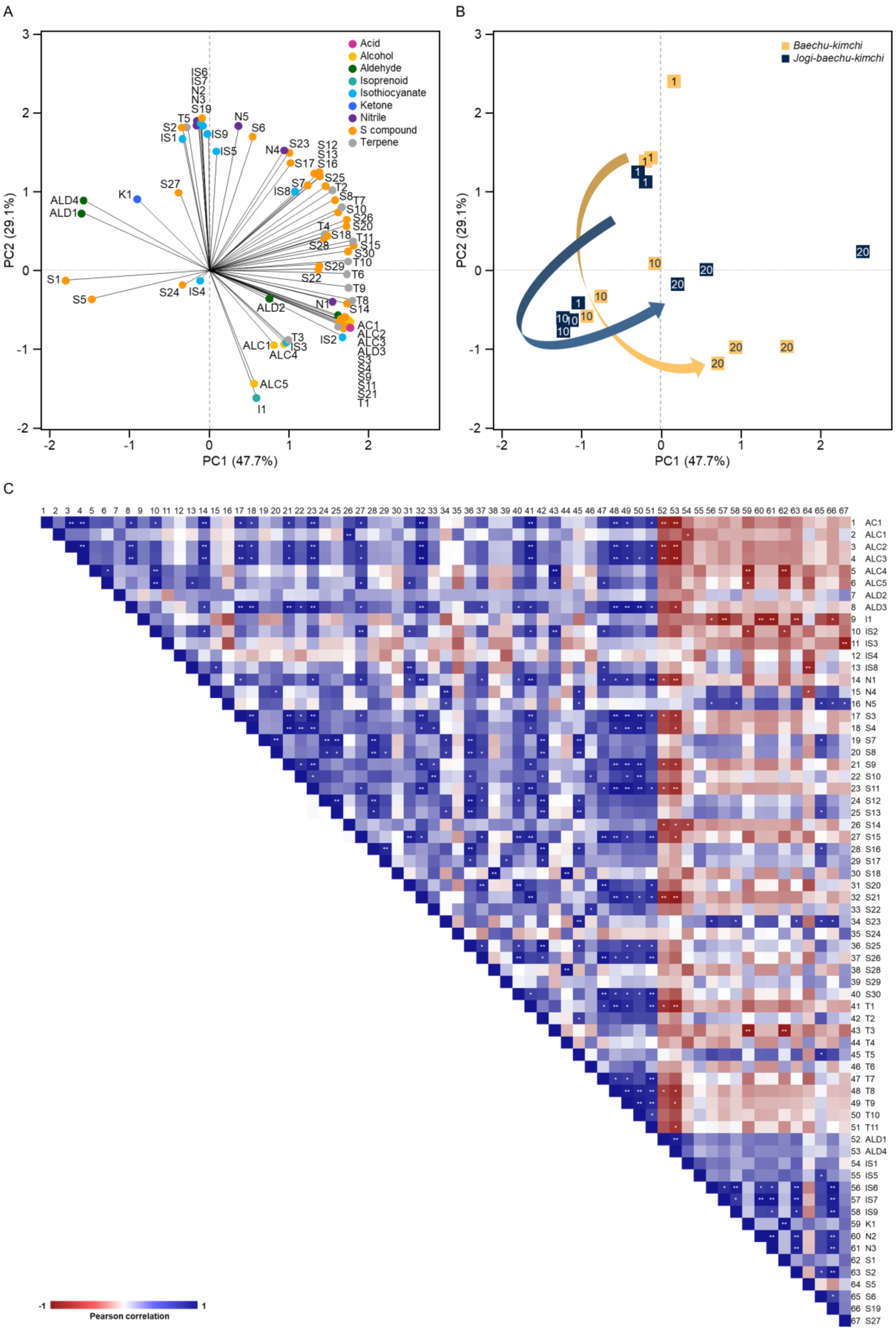

A
B
C
Acid
Alcohol
Aldehyde
Isoprenoid
Isothiocyanate
Ketone
Nitrile
S compound
Terpene
PC1 (47.7%)
PC2 (29.1%)
Baechu-kimchi
Jogi-baechu-kimchi
Pearson correlation
-1
1

Fig 2.

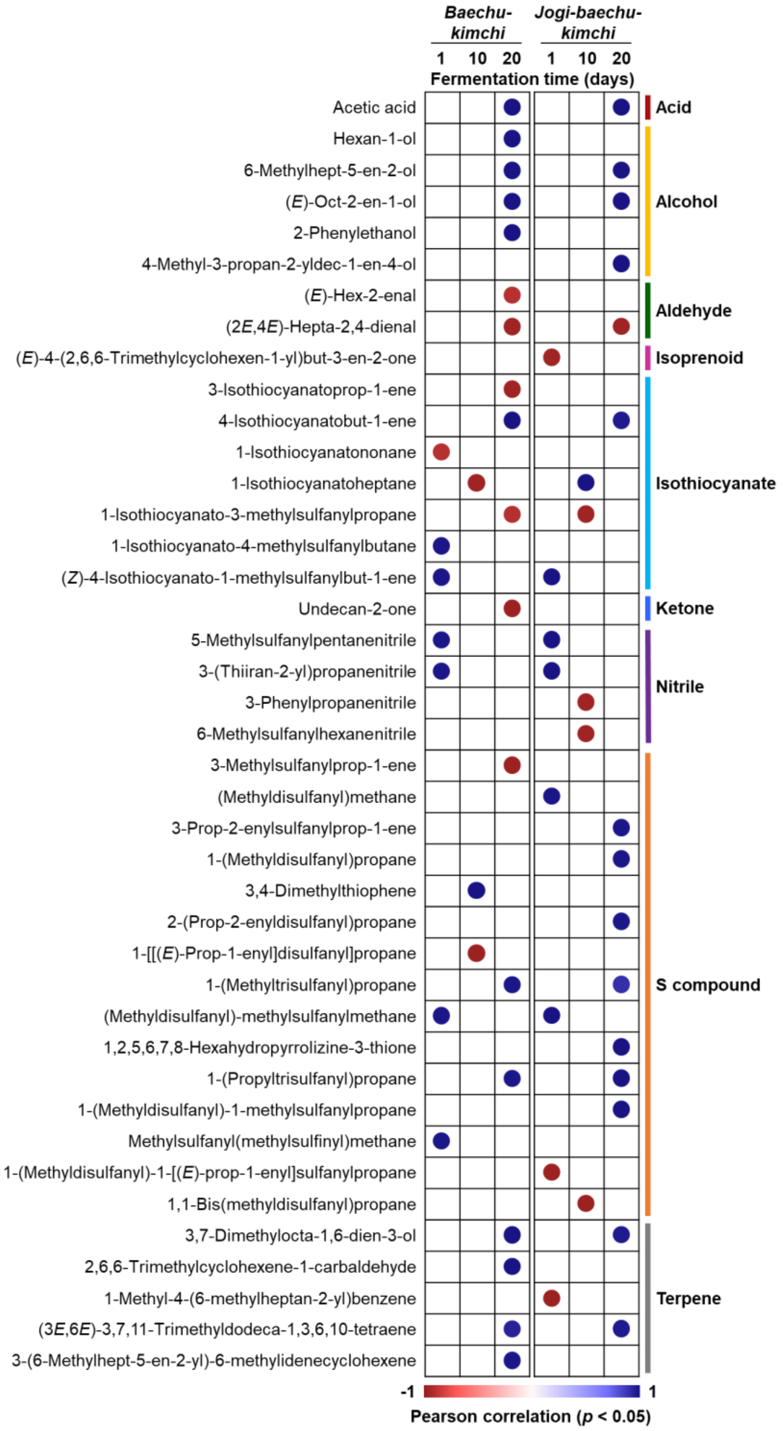
Baechu-kimchi
Jogi-baechu-kimchi
1 10 20 1 10 20
Fermentation time (days)
Acetic acid
Hexan-1-ol
6-Methylhept-5-en-2-ol
(E)-Oct-2-en-1-ol
2-Phenylethanol
4-Methyl-3-propan-2-yldec-1-en-4-ol
(E)-Hex-2-enal
(2E,4E)-Hepta-2,4-dienal
(E)-4-(2,6,6-Trimethylcyclohexen-1-yl)but-3-en-2-one
3-Isothiocyanatoprop-1-ene
4-Isothiocyanatobut-1-ene
1-Isothiocyanatononane
1-Isothiocyanatoheptane
1-Isothiocyanato-3-methylsulfanylpropane
1-Isothiocyanato-4-methylsulfanylbutane
(Z)-4-Isothiocyanato-1-methylsulfanylbut-1-ene
Undecan-2-one
5-Methylsulfanylpentanenitrile
3-(Thiiran-2-yl)propanenitrile
3-Phenylpropanenitrile
6-Methylsulfanylhexanenitrile
3-Methylsulfanylprop-1-ene
(Methyldisulfanyl)methane
3-Prop-2-enylsulfanylprop-1-ene
1-(Methyldisulfanyl)propane
3,4-Dimethylthiophene
2-(Prop-2-enyldisulfanyl)propane
1-[[(E)-Prop-1-enyl]disulfanyl]propane
1-(Methyltrisulfanyl)propane
(Methyldisulfanyl)-methylsulfanylmethane
1,2,5,6,7,8-Hexahydropyrrolizine-3-thione
1-(Propyltrisulfanyl)propane
1-(Methyldisulfanyl)-1-methylsulfanylpropane
Methylsulfanyl(methylsulfinyl)methane
1-(Methyldisulfanyl)-1-[(E)-prop-1-enyl]sulfanylpropane
1,1-Bis(methyldisulfanyl)propane
3,7-Dimethylocta-1,6-dien-3-ol
2,6,6-Trimethylcyclohexene-1-carbaldehyde
1-Methyl-4-(6-methylheptan-2-yl)benzene
(3E,6E)-3,7,11-Trimethyldodeca-1,3,6,10-tetraene
3-(6-Methylhept-5-en-2-yl)-6-methylidenecyclohexene
Acid
Alcohol
Aldehyde
Isoprenoid
Isothiocyanate
Ketone
Nitrile
S compound
Terpene
-1
1
Pearson correlation (p < 0.05)

Fig 3.

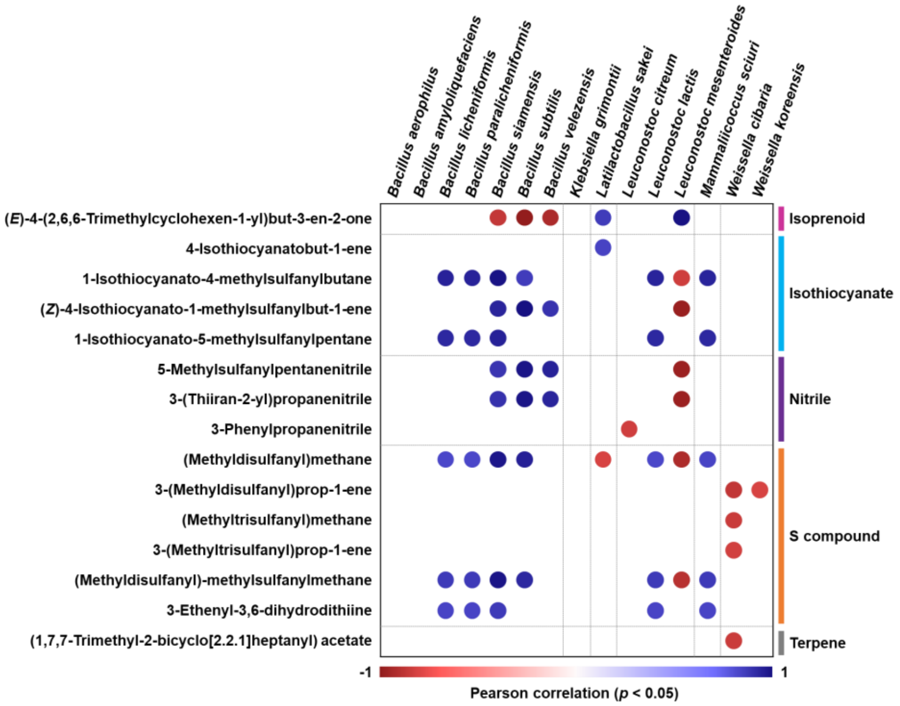
Bacillus aerophilus
Bacillus amyloliquefaciens
Bacillus licheniformis
Bacillus paralicheniformis
Bacillus siamensis
Bacillus subtilis
Bacillus velezensis
Klebsiella grimontii
Latilactobacillus sakei
Leuconostoc citreum
Leuconostoc lactis
Leuconostoc mesenteroides
Mammaliicoccus sciuri
Weissella cibaria
Weissella koreensis
(E)-4-(2,6,6-Trimethylcyclohexen-1-yl)but-3-en-2-one
4-Isothiocyanatobut-1-ene
1-Isothiocyanato-4-methylsulfanylbutane
(Z)-4-Isothiocyanato-1-methylsulfanylbut-1-ene
1-Isothiocyanato-5-methylsulfanylpentane
5-Methylsulfanylpentanenitrile
3-(Thiiran-2-yl)propanenitrile
3-Phenylpropanenitrile
(Methyldisulfanyl)methane
3-(Methyldisulfanyl)prop-1-ene
(Methyltrisulfanyl)methane
3-(Methyltrisulfanyl)prop-1-ene
(Methyldisulfanyl)-methylsulfanylmethane
3-Ethenyl-3,6-dihydrodithiine
(1,7,7-Trimethyl-2-bicyclo[2.2.1]heptanyl) acetate
Isoprenoid
Isothiocyanate
Nitrile
S compound
Terpene
-1
1
Pearson correlation (p < 0.05)

Fig 4.
