# Supplementary material for: Effects of jogi, Micropogonias undulatus, addition on the production of volatile compounds in baechu-kimchi
Source: PLoS One. 2024 Nov 18;19(11):e0312441. doi: 10.1371/journal.pone.0312441 (PMC11573143; doi:10.1371/journal.pone.0312441)
Supplement: Attachment — Submitted filename: 240904 Response to reviewers-PONE-D-24-29960-final.docx [file pone.0312441.s002.docx]

**PONE-D-24-29960 by Lee et al.**

**Itemized List of Changes**

***Responses to Reviewer #1***

Impact of addition of *jogi* on volatile compounds in kimchi were studies, however, it would be desirable to add details about its impact on taste and aroma? Add references, if already reported.

> Thanks for reviewer’s comments. As stated in the introduction, there have been very few comparative studies between the control group without the addition of seafood (especially *jogi*) and the group with its addition. Nevertheless, we have recently studied the effects of *jogi* addition on the microbial community (L70-77) and on the production of organic acids and amino acids (L77-83), and we have presented these findings along with the relevant references

L95: why sampling was done every 5 days ? should not based on previously experimentation of microbila communities dynamics? Or keep in view the major shift in composition? Moreover, results shown data at first, 10th and 20th day?

> According to the reviewer's comments, we have provided the kimchi fermentation days used in the experiment and the reasons for selecting those specific fermentation days (L95-99).

In general results presentation is good, but discuss lack rigor and comparison.

> The contents that should be in the discussion were divided into PCA and screening of volatile profiles of kimchi fermentation, Correlation of volatile compounds and fermentation periods, Correlation of volatile compounds and microbial community in kimchi sections and compared by the relevant contents.

Table 1; the values in decimals are relative abundence or quantificaiton ? no unit mentioned. Figure 1 B: how the amount was measured, nothing is given in materials and methods section

> Volatile compounds were identified using methyl cinnamate solution as the internal standard. The peak area of the internal standard and its value (μl) were quantified, and the total volatile compounds were quantified using a linear equation (L116-120).

Figure 2: title is not descriptive

> According to the reviewer's comments, we revised it (L270-271).

Figure 4; was correlations between individual microbes not the microbial communities and metabolic compounds, so title need correction.

> According to the reviewer's comments, we revised it (L343-344).

The new compounds were detected because of addition of *jogi* but nothing is disucssion the nature of metabolic pathways involved in the production of metabolities, what if these compounds were already present in *jogi* and slowly released during fermentation time ?

Any metabolic analysis of raw *jogi* is imparative to establish wheather the production was due to addition of *jogi* or its provide the precursers only?

> Thank you for your valuable comments. We are currently conducting research to determine whether the volatile compounds were originally present in the *jogi*, influenced by enzymes from the *jogi*, or resulted from the kimchi microorganisms. We have also added our suggestion in the manuscript (L251-255).

***Responses to Reviewer #2***

The manuscript entitled "Effects of *jogi*, *Micropogonias undulatus*, addition on the production of volatile compounds in *baechu-kimchi*" by Lee et al. intends to explore the effect of *jogi* addition on the volatile compounds presence in the kimchi. The manuscript is interesting and in the scope of Plos One, bringing new knowledge on the field of research. However, the methodology and Results and Discussion have some shortcomings. Detailed comments are provided in the highlighted text fragments.

> Thank you for your positive comments.

* Abstract

1. L27. “*Baechu-kimchi* without *jogi* was used as the control group.” This sentence is not necessary mention in the abstract.

> According to the reviewer's comments, we deleted it (L27).

2. L35 to 37. Please add Conclusions/significance in the abstract.

> According to the reviewer's comments, we added the conclusions (L36-38).

**Materials and methods

1. L 109. m/z italic

> We revised it (L113).

2. L115. Authors should add section for metagenomics analysis (based on previous published finding)

> The content of microbial community has been published as a previous study, so a reference paper has been added for that content (L124-126).

3. L 126 to 127. Include pre-processing step for GC-MS data prior to principal component analysis. Mention which software use to pre-process data. Include database used.

> According to the reviewer's comments, we added the software information (L114-115).

***Results and discussion

1. L163.

> According to the reviewer’s suggestion, we revised the S-compound and sulfur-compounds by matching them to sulfur compounds (L27, L40, L158, L164, L165, L170, L224, L227, L228, L260, L276, L335, L337, L338, Table 1, and Fig. 1-4).

2. L173. Redundant. This sentence was mentioned in the method.

> We deleted it (L180).

3. L232. Which day? for “Late-stages of fermentation”

> According to the reviewer's comments, we revised it (L239).

4. L239. Please standardize either to use S compounds or sulfur compounds.

> Changed to sulfur compound throughout the manuscript (L27, L40, L158, L164, L165, L170, L224, L227, L228, L260, L276, L335, L337, L338, Table 1, and Fig. 1-4).

5. L248 to 249. Include % of PC1 and PC2. Include statistical value R2 and Q2. Mention first which groups were separated by PC1 showing the variation of volatile compounds.

> Since Figure 2's PCA data (loading plot, Fig 2A; score plot, Fig 2B) already includes the percentages of PC1 and PC2, we did not specify them separately in the main text. Additionally, this study focused on observing the trends in the volatile compounds that increase or decrease throughout the fermentation process, rather than analyzing by specific categories of volatile compounds. As a result, it was somewhat challenging to distinctly separate the volatile compounds based on PC1. Therefore, we focused primarily on sulfur compounds, which were the most abundantly detected and showed clear changes between the early and late stages of fermentation.

6. L266 to 297. These abbreviations can be added in the Table 1 rather than write it in the text.

> According to the reviewer's comments, the abbreviation of Figure 2 is written in Table 1.

7. L 300. “fermentation times’

> According to the reviewer's comments, we revised it (L281).

8. L342 to 343. Relate this correlation with the pH changes based on the previous studies.

> Related content has been added to the pH change and the change in the volatile compound during the fermentation process (L354-358).

9. Should have conclusion section

> According to the reviewer's comments, Conclusion section divided separately (L363-377).

* Changes to the manuscript are indicated in red font.
